# Country, Cover or Protection: What Shapes the Distribution of Red Deer and Roe Deer in the Bohemian Forest Ecosystem?

**DOI:** 10.1371/journal.pone.0120960

**Published:** 2015-03-17

**Authors:** Marco Heurich, Tom T. G. Brand, Manon Y. Kaandorp, Pavel Šustr, Jörg Müller, Björn Reineking

**Affiliations:** 1 Department of Conservation and Research, Bavarian Forest National Park, Grafenau, Germany; 2 Chair of Wildlife Ecology and Management, University of Freiburg, Faculty of Environment and Natural Resources, Freiburg, Germany; 3 Van Hall Larenstein, University of Applied Sciences, Leeuwarden, The Netherlands; 4 Department of Biodiversity Research, Global Change Research Centre, Academy of Sciences of the Czech Republic, Brno, Czech Republic; 5 Šumava National Park, Vimperk, Czech Republic; 6 Chair for Terrestrial Ecology, Department of Ecology and Ecosystem Management, Technische Universität München, Freising, Germany; 7 Biogeographical Modelling, Bayreuth Center for Ecology and Environmental Research BayCEER, University of Bayreuth, Bayreuth, Germany; 8 UR EMGR Écosystèmes Montagnards, St.-Martin-d’Hères, France; University of Missouri Kansas City, UNITED STATES

## Abstract

The Bohemian Forest Ecosystem encompasses various wildlife management systems. Two large, contiguous national parks (one in Germany and one in the Czech Republic) form the centre of the area, are surrounded by private hunting grounds, and hunting regulations in each country differ. Here we aimed at unravelling the influence of management-related and environmental factors on the distribution of red deer (*Cervus elaphus*) and roe deer (*Capreolus capreolus*) in this ecosystem. We used the standing crop method based on counts of pellet groups, with point counts every 100 m along 218 randomly distributed transects. Our analysis, which accounted for overdispersion as well as zero inflation and spatial autocorrelation, corroborated the view that both human management and the physical and biological environment drive ungulate distribution in mountainous areas in Central Europe. In contrast to our expectations, protection by national parks was the least important variable for red deer and the third important out of four variables for roe deer; protection negatively influenced roe deer distribution in both parks and positively influenced red deer distribution in Germany. Country was the most influential variable for both red and roe deer, with higher counts of pellet groups in the Czech Republic than in Germany. Elevation, which indicates increasing environmental harshness, was the second most important variable for both species. Forest cover was the least important variable for roe deer and the third important variable for red deer; the relationship for roe deer was positive and linear, and optimal forest cover for red deer was about 70% within a 500 m radius. Our results have direct implications for the future conservation management of deer in protected areas in Central Europe and show in particular that large non-intervention zones may not cause agglomerations of deer that could lead to conflicts along the border of protected, mountainous areas.

## Introduction

The increasing human population combined with an increasing standard of living in many parts of the world have resulted in an increased exploitation of nature [1]. The natural areas that are left, such as protected national parks, can be seen as habitat islands in cultural landscapes, but are usually too small to accommodate all relevant ecological processes within park boundaries [2–5]. Most national parks are not large enough to sustain viable populations of large mammals, particularly those that engage in seasonal migration behaviour [6–8]. As a result, such animals also utilize landscapes surrounding protected areas. This could lead to conflicts as management objectives inside and outside protected areas can differ considerably. Inside national parks, the guiding principle is often the protection of ecological processes (natural process management), whereas outside the parks, management typically aims at optimizing recreational opportunities for hunters while minimizing complaints from farmers and foresters [10]. One likely cause of conflict outside protected areas could be high mammal densities inside protected areas [11–14].

Population measures, such as density or reproduction, are influenced by both the physical and biological environment as well as by human activities. Deer habitat selection is strongly determined by the presence of food and cover, both of which are correlated with forest distribution [15–17]; so too are ungulate density and forest structure [18]. Therefore, Gill, Johnson [19] suggest that forested areas may be one of the main factors that determine ungulate distribution. Variation in altitude has a major influence on local climate in mountainous environments, which is characterized by high precipitation, generally lower temperatures and long periods of snow coverage. High snow packs especially limit access to food and increase the energy needed for movement [17, 20, 21]. An important adaptation strategy of the animals is to migrate away from these climatic conditions towards lower elevations with less snow cover [7, 22].

Human conflicts with wildlife throughout Europe and North America often involve deer species owing to the dramatic increase in their populations over the last century [23–25]. This development was initially regarded positively, but opinion changed when overabundance led to a high economic impact because of bark peeling and increased browsing on forest vegetation and arable crops. As a consequence, ungulates attained an ambivalent status in society—watching or hunting the animals provides recreational pleasure, yet the animals are regarded as pests that cause considerable damage to agricultural fields and forests [26].

Hunting can be a key management factor for regulating deer populations and their spatial distribution in the landscape [27–29]. But whether hunting is effective strongly depends on local hunting regulations, philosophy, or hunter effort [30]. Even if the general objectives of hunting laws across Europe are similar, in practice, these laws can differ among countries because of differences in the regulation details and enforcement [31]. Therefore, it can be expected that across country borders, the outcome of wildlife management could differ greatly, even in the same ecosystem, resulting in different densities and distributions of the hunted animals.

Here we aimed at disentangling the effects of environmental variables and differences in management on the distribution of red deer (*Cervus elaphus*) and roe deer (*Capreolus capreolus*) in a Central European low mountain range, the Bohemian Forest Ecosystem. Using pellet counts, we specifically tested our predictions that relative distributional differences of red deer and roe deer are higher in the national parks, because of higher protection standards, higher in the Czech Republic due to a hunting policy that promotes high deer densities, and higher in the valleys since the animals leave the high ridges in winter. Additionally, we assume that red deer densities are higher in areas with more forest cover that provide protection from hunting, and roe deer densities are higher in areas with medium forest cover because of a trade-off between food availability and cover.

## Material and Methods

### Study area

The Bohemian Forest Ecosystem consists of a low, forested mountain chain approximately 130 km long and 60 km wide, situated along the border between Bavaria (Germany), the Czech Republic, and Austria. Elevation ranges from 370 m a.s.l. in the valleys up to 1,456 m a.s.l. along the mountain ridges. The climate is continental with some maritime influence from the west, with an annual precipitation varying from 400 mm to 2,500 mm. Permanent snow cover lasts up to 7 months on the mountain tops (October to May) and 5 months in the valleys (November to April). The centre of the area is formed by two contiguous protected areas, the Bavarian Forest National Park (240 km^2^) and the Šumava National Park (690 km^2^), which are surrounded by the Bavarian Forest Natural Park (3,007 km^2^) and the Bohemian Forest Protected Landscape Area (1,000 km^2^). The national parks are mostly forested, while their rural surroundings consist of smaller forests, meadows, arable land and villages. Compared to elsewhere in Europe, the human population density is very low, with 2 inhabitants/km^2^ in the core area, and at the margins (outside the national parks), approximately 70 inhabitants/km^2^ in Bavaria and 30 inhabitants/km^2^ in the Czech Republic.

Roe deer, red deer and wild boar (*Sus scrofa*) are widely distributed in the area, while moose (*Alces alces*) is found only in small numbers in the southern part. The only large predator is Eurasian lynx (*Lynx lynx*), which was reintroduced in the 1970s and 1980s [32]. At present, this lynx population is stagnant [33]. Lynx prey mainly on roe deer and to a much lesser extent on red deer [34].

**Fig 1:**
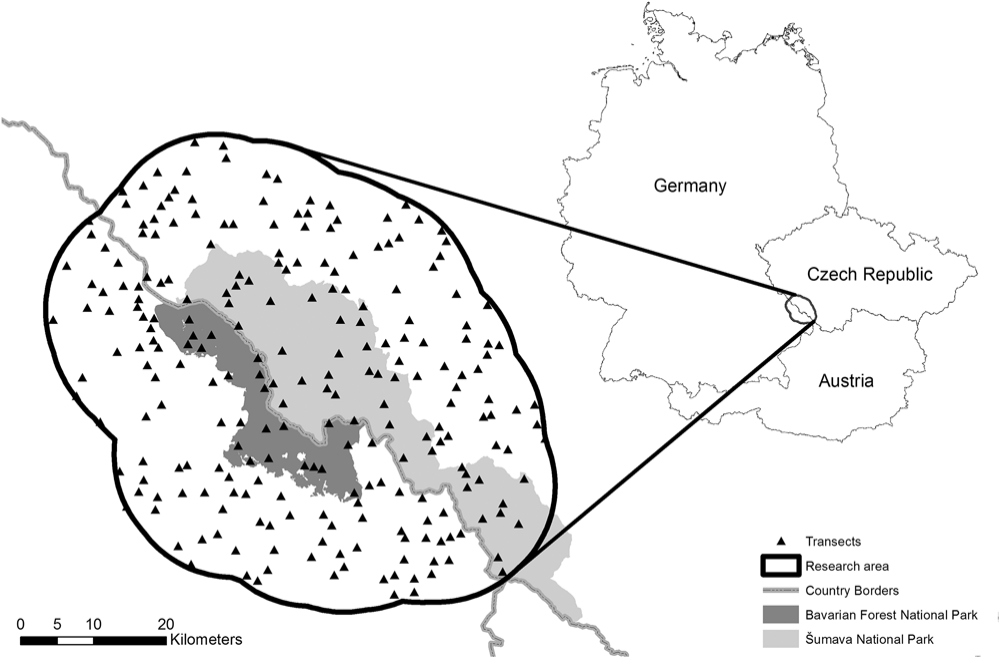
Overview of the study area. The locations of the national parks and transects used for pellet group counting are shown.

Our analyses of the Bohemian Forest Ecosystem included a 15 km wide buffer zone around the Bavarian Forest National Park and the northern part of the Šumava National Park. The entire study area covered 3,354 km^2^ (Fig. 1).

### Wildlife management

The priorities of game management in Bavaria and the Czech Republic considerably differ. Bavaria focuses on the natural regeneration of forests, and the Czech Republic focuses more on hunting.

In Bavaria, hunting, forestry, and state forest law, natural forest regeneration has priority over wildlife. The aim is to change the silvicultural system towards near-natural forests, with a shift from coniferous forest to mixed forest that relies on natural forest regeneration. Therefore, ungulate densities have to be kept at a level that allows natural regeneration of the main tree species without protective measures [35, 36]. The traditional ungulate census was shown to be inaccurate and was replaced by state-wide regeneration surveys every three years, which now are the foundation for setting hunting quotas [27]. In Germany, hunters have to pay compensation to landowners for damage caused by ungulates, and landowners are sensitized to such damage [37].

In the Czech Republic, management objectives are formulated in the Game Management Act (2003) for the maintenance of sustainable numbers of game, prevention of damage, maintenance of game quality and genetic purity. The hunting ground user is responsible for controlling game numbers and damage compensation. Wildlife management is hunter dominated and traditional, with selective hunting of old and sick animals, underdeveloped young and trophy animals. The hunting quotas are based on a visual survey performed by the hunters in spring without any monitoring of wildlife impact on the environment [38]. Since compensation for damages in forestry and crop production by the hunting ground users is rarely claimed or paid, hunters are not pressured to limit deer numbers [38].

Outside the national parks on both sides of the border, regular hunting and intense winter feeding occurs. In contrast, in the national parks the principal aim is to reduce intervention as much as possible. A roe deer non-intervention zone covering 89,000 ha has been established, and no winter feeding takes place. Red deer have a continuous non-intervention zone comprising 23,000 ha, but in winter, the animals are fed within the parks in enclosures [29].

In Germany, the range of red deer outside the park is restricted by law. Animals are not allowed to migrate to their natural winter habitat because these areas are outside the designated red deer management area. Therefore, in the 1970s, the national park managers constructed four winter enclosures encompassing a 30–50 ha fenced area with a central feeding place. After the rutting period in October when the first snow falls, red deer move to the enclosures. Animals arriving later are trapped in small pre-enclosures (less than 2 ha), and this entire group is then either led to the main enclosures for the winter or culled. Eighty per cent of shot red deer are killed in this way. In the beginning of May after the flush of ground vegetation, the enclosures are opened. The purpose of this management measure is to compensate for the restricted winter habitat, to simulate the winter absence of the species in the montane forest, and to control the population [39, 40]. Winter enclosures are also maintained in the Šumava National Park (N = 13), but no animals in the enclosures are culled. About two-thirds of the red deer herd overwinter in the enclosures.

### Pellet counts

Pellet group counts were permitted by the authorities of Šumava National Park and Bavarian Forest National Park. Outside of the parks, landowner permission was obtained. We confirm that the field studies did not involve endangered or protected species. There was no approval by an animal ethics committee necessary, because sampling was not invasive and did not disturb animals.

**Fig 2:**
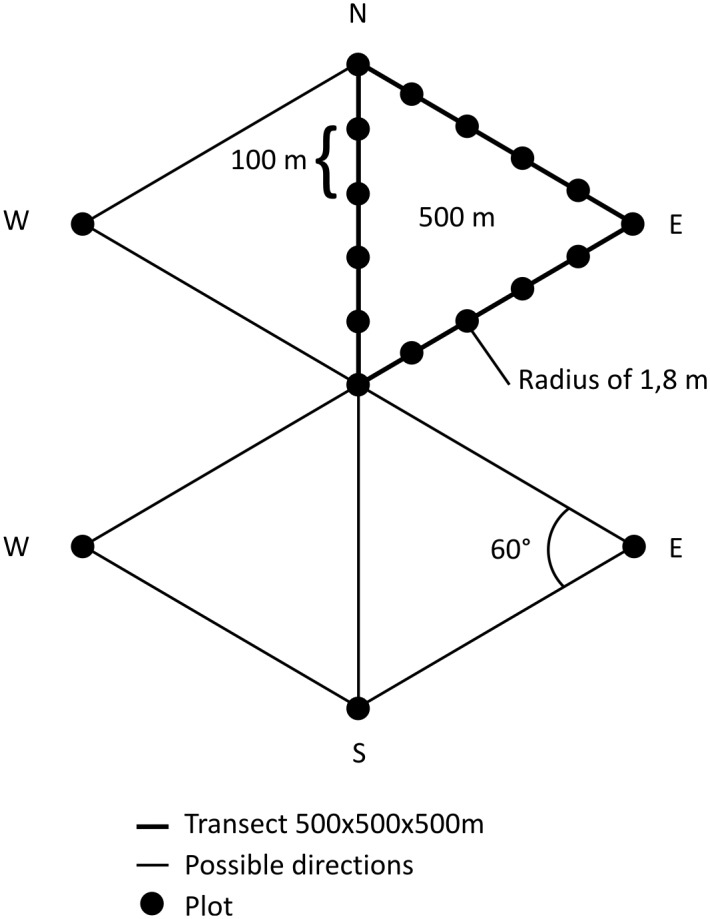
Design of triangular transects sampled for pellet group counts.

We examined the spatial distribution patterns of red deer and roe deer using the standing crop count method based on counts of pellet groups. We sampled the study area on circular plots along triangular transects [17, 41]; 218 randomly distributed triangular transects were set up, with a minimum target distance between transect centres of 1,000 m (Fig. 1). Starting at the determined coordinates, we walked along equilateral triangular transects of 1,500 m following GPS bearings (Garmin eTrex Vista HCx). Every 100 m, a plot with a radius of 1.8 m (10 m^2^) was sampled (15 plots per triangular transect; Fig. 2). Transects were oriented towards the north and east–south–east, except when it was not possible to walk north and/or east because of a village, river or other obstructions. Transects were not sampled when the start location was not accessible or when the transect was located within a village. No transect was located in a winter enclosure. Pellet groups were counted only if the group had at least two pellets. If a pellet group was lying on the plot border, the count was included only if at least two pellets were within the plot. Red deer and roe deer pellets were differentiated by their size (roe deer pellets are half the size of red deer pellets) and shape (roe deer pellets are rounder). The survey was conducted following snow melt, between 7 April and 12 May 2010, starting at lower elevations [42–45].

To obtain an indicative estimate of pellet decay rates, we exposed 24 pellet groups of each species on four different dates (30 April, 25 June, 20 August, 20 October 2010). The exposed pellet groups were checked every two weeks from April to October 2010 and once in April 2011.

### Explanatory variables

The mean elevation of plots within transects was taken from the Shuttle Radar Topography Mission (SRTM) with 90 m spatial resolution [46]. Land cover was calculated from CORINE Land Cover 2008 at a scale of 1:100,000 (http://www.eea.europa.eu/). For each transect, the fraction of land cover classified as forest or as human dominated was calculated within a buffer zone of 500 m around the transect centre. Human-dominated land cover corresponded to CORINE classes 1–11; forest cover corresponded to CORINE classes 23–25 and 27–34. In addition, we used the following explanatory variables: whether a transect was within a national park, country in which the transect was located, interaction of national park and country.

We assessed the extent of collinearity between explanatory variables; absolute values of the Pearson correlation coefficient were < 0.6 for all pairs of explanatory variables, which is below the recommended threshold of 0.7 [47].

### Data analysis

In the analysis, triangular transects were used as sample units; pellet groups found per plot within transects were summed for each species. The number of pellets was modelled for each species separately. As explanatory variables, we included the two categorical variables country and national park as well as their interaction; the continuous explanatory variables fraction of forest cover within a 500 m buffer zone and elevation were included with quadratic terms.

For each species, we followed the same modelling strategy. All models accounted for overdispersion by using a negative binomial family. First, we investigated whether accounting for zero inflation improved model performance as measured by AIC. We compared four models, each with the same model structure for the count data, i.e. the full model with both categorical variables and their interaction as well as the two continuous variable with quadratic terms: a generalized linear model, and three zero-inflated negative binomial models where the zero inflation was modelled by either a constant, a linear function of elevation, or a quadratic function of elevation. Models where the zero inflation was modelled with country and national park, with or without interaction term, did not converge. Second, we tested for spatial autocorrelation in the residuals with Moran’s I for distances up to 15 km. If significant spatial autocorrelation was found, we accounted for this by using generalized linear mixed models with an exponential spatial correlation structure, i.e. the correlation in the residuals between transects is modelled to decline exponentially with the distance between transects [48]. For this, all transects were assigned to the same group, i.e. there are no separate independent random effects for each transect. Third, explanatory variables were selected using backward stepwise selection with AIC as selection criterion. Variable importance was assessed with a randomization procedure [49]. Each explanatory variable in turn was randomized 100 times, and for each randomization a model prediction of the pellet counts was made. The raw importance value for each explanatory variable was calculated as one minus the mean correlation between predicted pellet counts using the original and the randomized explanatory variables. These raw importance values were then normalized to a sum of one.

Approximate point-wise 95% confidence intervals of model predictions were computed from 1,000 bootstrap samples using an ordinary bootstrap for the non-spatial zero-inflated negative binomial model for red deer and a grid-based block bootstrap [50] for the generalized linear mixed model (GLMM) with spatial autocorrelation correlation structure for roe deer. For the grid-based block bootstrap, we divided the study region into 5 (north–south direction) × 4 (west–east direction) rectangular blocks, with a size of ca. 12,500 m (north–south) × 16,500 m (west–east). Grid points were created by subdividing the rectangular blocks in 10 × 10, i.e. 100 smaller rectangles. The blocks were clipped to the convex hull of the transect locations. For each bootstrap sample, each block was randomly shifted to one of the grid points; we only selected grid points where at least 95% of a block’s area was within the convex hull of the transect locations. The transects that were covered by a shifted block were selected for that bootstrap sample and were moved inside the area originally covered by the shifted block by adding the difference between the block’s original lower-left coordinates and the grid point’s lower-left coordinate.

Data were statistically analysed using R version 2.13.0 [51]; zero-inflated negative binomial models were fitted with function zeroinfl [52] from package pscl version 1.4.6 [53]; GLMMs were fitted with package mgcv version 1.7–5 [54].

## Results

The pellet group counts of red deer and roe deer differed substantially. Roe deer pellet groups were more abundant and more widely distributed. On 117 of the 218 transects sampled, a total of 658 red deer pellet groups were found, whereas on 156 transects, a total of 1,069 roe deer pellet groups were found.

The persistence probability of red deer pellets was much higher than for roe deer pellets: 96, 76, 38.1 and 11.5% of red deer pellet groups that were exposed in October, August, June and April, respectively, were found in April 2011, whereas 70.8, 5.3, 4.6 and 1.3%, respectively, of roe deer pellet groups were found in April 2011.

The distribution of both red deer and roe deer could be explained to a similar extent with the chosen predictor variables; the final models for red deer and roe deer had an adjusted R^2^ of 0.26 for red deer and 0.34 for roe deer, respectively (Tables 1 and 2). The relative importance of predictor variables and functional relationships differed between the two species (Table 3).

**Table 1:** Summary of the final zero-inflated model for predicting numbers of red deer pellet groups.

Count model coefficients (negative binomial family with log link)					
	Parametric coefficients				
Variables	Estimate	Std. error	t-value	Pr(>|t|)	
(Intercept)	−0.1701	0.2041	0.833	0.405	
Poly(forest,2)1	3.957	1.669	2.370	0.018	*
Poly(forest,2)2	−5.714	1.4781	−3.866	0.000	***
National park	1.354	0.313	4.321	0.000	***
Country (Czech Republic)	1.922	0.239	8.061	0.000	***
National park:Country (Czech Republic)	−1.446	0.359	−4.034	0.000	***
Log(theta)	0.508	0.203	2.502	0.012	*
Zero-inflation model coefficients (binomial family with logit link)					
Variable	Estimate	Std. error	t-value	Pr(>|t|)	
(Intercept)	−5.725	2.571	−2.227	0.026	*
Poly(elev,2)1	−109.929	47.098	−2.334	0.0196	*
Poly(elev,2)2	−51.150	25.567	−2.001	0.045	*
R-sq.(ad):0.26					

Sample size (number of transects): 218. Red deer ~ poly(forest, 2) + country * national park | poly(elev, 2). Significance codes: 0 '***' 0.001 '**' 0.01 '*' 0.05 '.' 0.1 ' ' 1.

**Table 2:** Summary of the final generalized linear mixed-effects model with a negative binomial family for predicting numbers of roe deer pellet groups and exponential spatial error structure.

	Parametric coefficients				
	Estimate	Std. error	t-value	Pr(>|t|)	
(Intercept)	0.274	0.281	0.975	0.331	
Forest	0.832	0.307	2.710	0.007	**
Poly(elev,2)1	-9.645	1.849	-5.217	0.000	***
Poly(elev,2)2	-3.201	1.465	-2.185	0.030	*
National park	-1.123	0.277	-4.051	0.000	***
Country (Czech Republic)	1.165	0.198	5.862	0.000	***
R-sq.(ad):0.34					

Sample size (number of transects): 218. Roe deer ~ forest + poly(elev,2) + country + park. Significance codes: 0 '***' 0.001 '**' 0.01 '*' 0.05 '.' 0.1 ' ' 1.

**Table 3:** Percentage of variable importance in the final selected zero-inflated negative binomial model of red deer and generalized linear mixed-effects model of roe deer.

	Variable importance (%)	
Variable	Red deer	Roe deer
Forest	18.8	9.9
Elevation	26.9	33.1
Country	49.2	40.2
National park	5.1	16.8
Total	100	100

The variable forest is the percentage of forest within a 500 m radius around the centre of the triangular transects. Elevation is in m a.s.l.

For red deer, the best model according to AIC was a zero-inflated negative binomial model where zero inflation was modelled by a quadratic term of elevation. There was no indication of spatial autocorrelation in the residuals of red deer for the range of tested spatial lags. For roe deer, the best model according to AIC was a negative binomial model; however, the difference in AIC to the best zero-inflated negative binomial model, which was the one without any explanatory terms, was low (ΔAIC < 2). All models for roe deer had statistically significant residual spatial autocorrelation. We therefore used a GLMM with spatial autocorrelation term; the estimated range for the exponential spatial correlation was 1,082 m.

Our first prediction was that red deer and roe deer distribution would be more influenced by the national parks because of higher protection standards. However, significantly more roe deer pellet groups were found outside the national parks (β_National park_ = –1.1, SE = 0.28, t = –4.05, P < 0.001) (Table 2). For red deer, the influence of the national parks differed between the parks. Šumava National Park had no significant effect on the number of red deer pellet groups found in the Czech Republic. In Bavaria, the number of pellet groups was significantly higher within the Bavarian Forest National Park (β_National park_ = 1.35, SE = 0.31, t = 4.32, P < 0.001) than outside the national park (Table 1). The variable national parks was the least important factor considered in explaining the distribution of red deer (5.1%) and the second-least important factor of four factors explaining the distribution of roe deer (16.8%) (Table 3).

In agreement with our second prediction that red deer and roe deer distribution was significantly influenced by country, both red deer and roe deer pellet groups were more abundant in the Czech Republic (red deer: β_Czech Republic_ = 1.92, SE = 0.24, t = 8.06, P < 0.001; roe deer: β_Czech Republic_ = 1.16, SE = 0.20, t = 5.86, P < 0.001) (Tables 1 and 2). The variable country had the greatest influence on the distribution of both species (red deer: 49.2%; roe deer: 40.2%) (Table 3).

**Fig 3:**
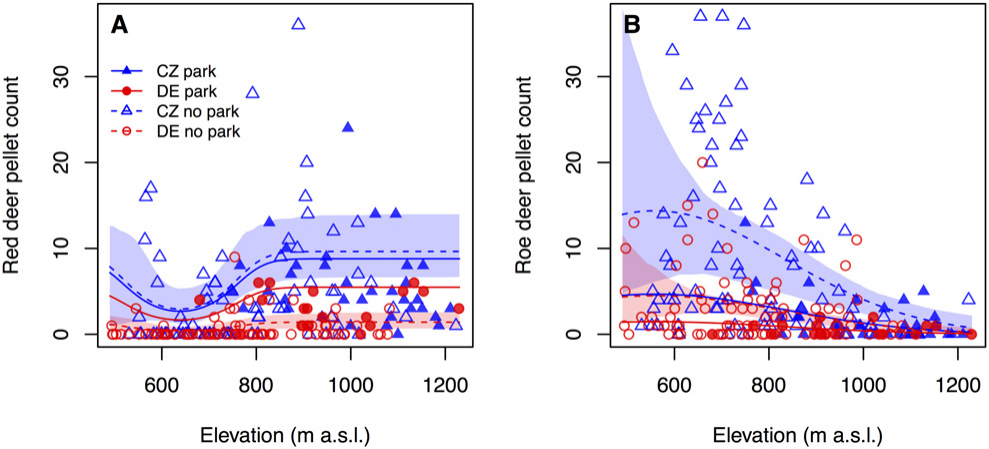
Effect of elevation on the number of deer pellet groups. Shaded areas indicate bootstrapped point-wise 95% confidence intervals; confidence intervals are only shown for areas outside national parks to improve readability. A) Red deer pellet groups; model parameters are provided in Table 2. B) Roe deer pellet groups; model parameters are provided in Table 3.

In agreement with our third prediction, elevation had a significant influence on the distribution of both red deer (X^2^ = 44.62, df = 2, P < 0.001) and roe deer (df = 2, F = 14.05, P < 0.001) (Tables 1 and 2). The variable elevation had a greater influence on roe deer distribution than on red deer distribution (red deer: 26.9%; roe deer: 33.1%; Table 3). In contrast to our prediction, the probability of finding red deer pellet groups was lowest in areas at about 640 m a.s.l. and increased towards both lower and higher elevations (Fig. 3A). The highest density of roe deer pellet groups was found at 560 m a.s.l. and decreased continuously with increasing elevation (Fig. 3B).

**Fig 4:**
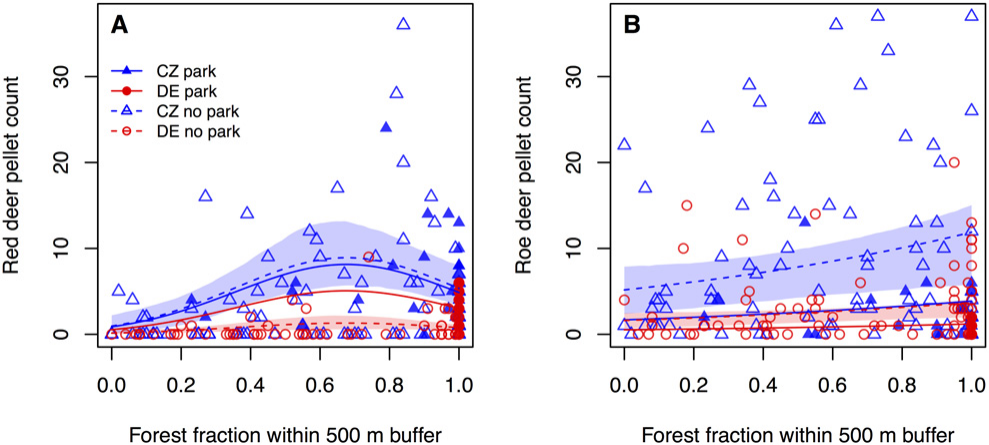
Effect of forest cover on the number of deer pellet groups. Shaded areas indicate bootstrapped point-wise 95% confidence intervals; confidence intervals are only shown for areas outside national parks to improve readability. A) Red deer pellet groups; model parameters are provided in Table 2. B) Roe deer pellet groups; model parameters are provided in Table 3.

Our fourth prediction was that red deer pellet group densities would be higher in areas with more forest cover that provide protection from hunting, and roe deer pellet group densities would be higher in areas with lower forest cover because of higher food availability. Our results indicated that the amount of forest in the area of the transect significantly influenced the number of both red deer pellet groups found (X^2^ = 17.38, df = 2, P < 0.001) and roe deer pellet groups found (β_Forest_ = 0.83, SE = 0.31, t = 2.71, P < 0.01) (Tables 1 and 2). Forest had a stronger influence on the number of red deer pellet groups (18.8%, Fig. 4A and Table 3) than on the number of roe deer pellet groups (9.9%, Table 3) and therefore a better defined effect on red deer. The number of red deer pellet groups increased up to about 70% forest cover, and declined thereafter. The relationship between the number of roe deer pellet groups and forest cover was linear on the log scale and slightly positive (Fig. 4B).

## Discussion

Our analysis of the number of roe deer and red deer pellet groups yielded new information on the potential drivers of ungulate winter distributions in the areas surrounding a protected area in Central Europe. The results showed that environmental and management-related factors are equally important. In contrast to our predictions, the national parks had no strong positive effect on the distribution of red deer and roe deer. The effect of country was much stronger, with a higher pellet group density of both species found in the Czech Republic. In line with previous studies [7, 22], elevation had a negative effect on the probability of finding roe deer pellet groups, while the probability of finding red deer pellet groups increased both above and below 640 m. Forest cover had, as expected, a stronger positive effect on red deer than on roe deer pellet group density.

Because our study examined the relative distributional differences of red deer and roe deer and not absolute numbers, pellet counting was deemed to be the most cost-effective and comprehensive method for this large-scale research, which would need to be repeated in the following years to function as a management evaluation tool [55]. Despite its low costs and practicability, this indirect observation method has been shown to provide reliable results in earlier studies [17, 56–59]. We chose the standing crop count method because results are similar to those of the clearance plot method, but the effort is much lower [60, 61]. Additionally, this pellet count method is applicable in areas where animal densities are low, because it allows enough time for pellet groups to accumulate [56]. We counted the pellets directly after snow melt as this allows the pellet groups to build up over winter, when pellets are not decomposed by invertebrates [43, 62]. Moreover, the sparse vegetation during that time allows easy detection of the pellets [63]. However, the decay of red deer pellet groups was much slower than were the decay of roe deer pellet groups. Therefore, the results of this study show the winter distribution of roe deer and the late summer to winter distribution of red deer, which has to be considered in the interpretation of the results.

In our study, we did not account for different pellet decay rates, which might be of importance because of the large altitudinal gradient sampled. Even if the pellets are sampled directly after snow melt, the accumulation time between the highest and lowest altitudes differs because of differences in the time period with snow coverage. Therefore, to interpret our results, we have to consider that we possibly overestimated deer densities at the higher elevations because pellet decay rates there are likely lower. We also did not account for different defecation rates, which might depend on the population structure of the ungulate population and the quality of available forage [64, 65]. It is well known that food quality can influence the digestion of herbivores. Therefore, pellet surveys should be used with caution in areas where the quality of forage varies strongly in the landscape [55]. Food analysis of red deer [66] and roe deer [67] in the Bohemian Forest Ecosystem show that generally red deer are mixed feeders and roe deer are browsers. The predominant food of both species comes from forests as well as meadows and pastures. The amount of arable land in the study area is low, and therefore high-energy food can hardly be found in the diet. The differences in food quality should therefore be low within the study area. Also, there are no indications of differences in population structure across the study area.

A major finding of our study was the small effect of the national parks on the distribution of the animals. We assumed that deer pellet counts would be lower outside of the protected areas than in protected areas with less or no intervention. However, the differences in the number of pellet groups inside and outside national park boundaries suggested that the national parks do not have a major influence on deer distribution. The influence of the national parks on roe deer winter distribution was even negative instead of the expected positive effect, although the non-intervention zone covers 89,000 ha. Likely reasons for these results are the closure of feeding stations, in combination with high snow packs, high forest cover and lynx presence in the national parks. Before the establishment of the Bavarian Forest National Park in the beginning of the 1970s, about 40 feeding stations were placed in the Rachel-Lusen area (130 km^2^). These feeding stations were successively closed each year until the last was closed in 1985, whereas feeding outside of the park increased during this period and has remained high since then. As a consequence, the natural migration of roe deer to wintering habitats outside of the national park was re-established [22]. Also the forest cover within the parks is higher than in the outside areas, which leads to a better roe deer habitat in the foothills of the parks, where meadows of high nutritional value for roe deer are interspersed in the forests [67]. Moreover, roe deer are the preferred food source for Eurasian lynx in the study area [34], and lynx has a significant negative impact on the survival of roe deer [68]. The lynx density is about 1 individual per 100 km^2^ inside the national parks and about 0.5 individuals per 100 km^2^ outside the national parks [69]. The increased predation caused by the higher lynx density inside the national parks could contribute to lower roe deer density in these areas. In contrast, red deer density was higher in the Bavarian Forest National Park than in its surroundings. Red deer are heavily hunted in the designated red-deer-free areas in Bavaria to facilitate natural forest regeneration. Also control measures within the winter enclosures in the Bavarian Forest National Park limit the red deer density to a lower level [29]. In the Czech Republic, the red deer density inside and outside the national park is equally high. The reason for this is twofold: first, the red deer range is not restricted there and the animals can range freely also outside the national park, and second, the winter enclosure system is not applied as consistently as in Bavaria. In the Czech Republic, only about 50% of the animals stay in enclosures, while in Bavaria, more than 80% do so. Because the animals staying in winter in the enclosures are not represented by the applied method, the relative distributional differences of red deer in the Šumava National Park indicated by the pellet counts is probably overestimated in comparison to Bavarian Forest National Park. From our results, we can conclude that the current park management neither on the Bavarian side nor on the Czech side of the border contributes to higher deer densities in privately owned forests outside the parks.

The most important factor influencing the distribution of the animals in the Bohemian Forest Ecosystem was the country, with a higher pellet density of both species in the Czech Republic. This difference could possibly be explained by several factors, such as different topography (mean height above sea level), different land-cover types and different game management. A higher mean height above sea level would lead to a slower decay of deer pellets and migration of roe deer out of the areas in winter. As the mean height of sample plots in the Czech Republic (839 m) are higher than those in Bavaria (796 m), the opposite results, i.e. higher pellet counts in Bavaria, would have been expected. Therefore, topography is not the reason for the effect of country. In our study area the Bavarian part has a higher forest cover (79%) than the part of the Czech Republic (65%); therefore, conditions for red deer and roe deer should be better in Bavaria. Although one would expect that the more intense agriculture and higher human population density in Germany would lead to lower deer populations, a study has shown that roe deer can reach high population densities in areas with a much higher human population density and a much more intense agriculture [70] than in our study area. Also red deer should be able to cope with these conditions in Bavaria particularly, because of the large forested areas that allow the animals to find protective cover and forage. Therefore, also land-cover type is not the reason for the effect of country on deer populations. We attribute the differences in Bavaria, Germany and the Czech Republic mainly to the different hunting policies in these two countries. At first glance, the hunting systems are similar, with hunting rights belonging to the landowner, the area divided into districts, and the hunters taking responsibility for damages incurred by game species. But in practice, the systems differ. In the Czech Republic, financial compensation given to farmers and forest owners for damage caused by ungulates is relatively low, and the hunting system is oriented toward the objectives of the hunters, who are not obligated to control the ungulate populations [38]. In contrast, in the attempt of the Bavarian government to change forests from coniferous plantations to mixed forests, both the Bavarian Forest and the hunting laws aim at maintaining free-living ungulates at low densities that allow unfenced natural forest regeneration of locally abundant tree species [35, 36]. To reach this objective, a state-wide monitoring system for forest regeneration has been established and hunting quotas are based on the results of this monitoring system. This system has already proven to be successful for the reduction of deer densities and browsing pressure at the state level [27] and might also explain the differences observed in the Bohemian Forest Ecosystem.

In accordance with our expectations, we found a negative linear relationship between counted roe deer pellet groups and elevation. This pattern is caused by seasonal migrations that lead to a concentration of deer in the valleys in winter, a pattern also observed in other areas with a distinct seasonality [6, 71]. Elevation was the second most influential factor of roe deer and red deer distribution. However, the effect of elevation on red deer was not as predicted. The lowest red deer pellet group density was at 640 m and densities were higher at lower and higher elevations. Also, the influence of elevation on red deer was lower than the influence on roe deer. We attribute this difference to the different management of red deer and roe deer. First, roe deer are not fed in the national parks, and the animals follow their migration routes without being stopped. In contrast, red deer are fed in winter enclosures, a management tool for reducing browsing impact in winter by luring the animals to feeding stations enclosed with a fence. After the first animals have migrated into the enclosure, the gates are closed. Animals that appear later are caught and fed in a pre-enclosure and are then led into the main enclosure or, in Germany, possibly culled instead. Consequently, the animals roam for a long period in the vicinity of the enclosures before they are trapped, which leads to a higher density there at elevations of about 900 m a.s.l. [72, 73]. Second, in Bavaria, red deer that migrate to traditional winter ranges at lower elevations outside of the national park will be shot due to zero-tolerance policy for red deer outside the designated red deer areas [35]. This policy contributes to the lower chance of finding red deer pellets at elevations of about 640 m. Moreover, the differences between red deer and roe deer pellet counts are also caused by different decay rates, with roe deer pellets decaying faster than red deer pellets. Therefore, the counted roe deer pellets represent the winter distribution, while a considerable amount of red deer pellets also originate from late August and September, when the animals live at the higher elevations.

Habitat selection of both red deer and roe deer was significantly influenced by the amount of forest, consistent with our prediction. The presence of forest cover was the second most important factor explaining the distribution of red deer, but the least important factor explaining the distribution of roe deer. The increasing preference of red deer for forest cover peaked at 70% coverage, which corresponded with the results of Prokešová [56]. In the study of Borkowski and Ukalska [17], red deer also showed higher requirements for cover than roe deer. The question arises why the influence of forest cover differs between the two deer species. If we consider feeding behaviour, we would not expect red deer to graze in areas with high percentages of forest cover. A more likely reason is that red deer avoid areas with high hunting pressure and high human population densities and seek refuge in larger forests [74]. Moreover, the natural spruce forests in the study area offer a large food supply of grasses, which can be utilized without leaving the forest [75]. In contrast, roe deer is an ecotone species that prefers landscapes with smaller, fragmented forests and a high amount of forest–meadow edges habitat to fulfil their food requirements [76, 77]. Therefore, forest cover is the least important variable for this species; however, the number of pellets found increases slightly with forest cover.

Based on our empirical data on deer distribution, we propose the following implications for the future conservation management of deer in protected areas in Central Europe. First, under certain preconditions it is possible to allow large non-intervention zones without causing deer to agglomerate, which could lead to conflicts along the border of protected areas. Second, factors related to national game management can strongly impact the wildlife within protected areas, even if international guidelines [9] require that active management within national parks should be minimized.

## Supporting Information

S1 Dataset Data table. (ZIP)
